# A low-cost quantitative continuous measurement of movements in the extremities of people with Parkinson's disease

**DOI:** 10.1016/j.mex.2018.12.017

**Published:** 2019-01-04

**Authors:** Gregory Neal McKay, Timothy P. Harrigan, James Robert Brašić

**Affiliations:** Johns Hopkins University, Baltimore, MD, United States

**Keywords:** Quantitative continuous movement measurement space, Accelerometer, Instrumentation, Signal processing

## Abstract

The assessment of Parkinson’s disease currently relies on the history of the present illness, the clinical interview, the physical examination, and structured instruments. Drawbacks to the use of clinical ratings include the reliance on real-time human vision to quantify small differences in motion and significant inter-rater variability due to inherent subjectivity in scoring the procedures. Rating tools are semi-quantitative by design, however, in addition to significant inter-rater variability, there is inherent subjectivity in administering these tools, which are not blinded in clinical settings. Sophisticated systems to quantify movements are too costly to be used by some providers with limited resources. A simple procedure is described to obtain continuous quantitative measurements of movements of people with Parkinson’s disease for objective analysis and correlation with visual observation of the movements.

• Inexpensive accelerometers are attached to the upper and lower extremities of patients with Parkinson’s disease and related conditions to generate a continuous, three-dimensional recorded representation of movements occurring while performing tasks to characterize the deficits of Parkinson’s disease.
• Movements of the procedure are rated by trained examiners live in real-time and later by videotapes.
• The output of the instrumentation can be conveyed to experts for interpretation for diagnostic and therapeutic purposes.

## Graphical abstract

**Figure:**
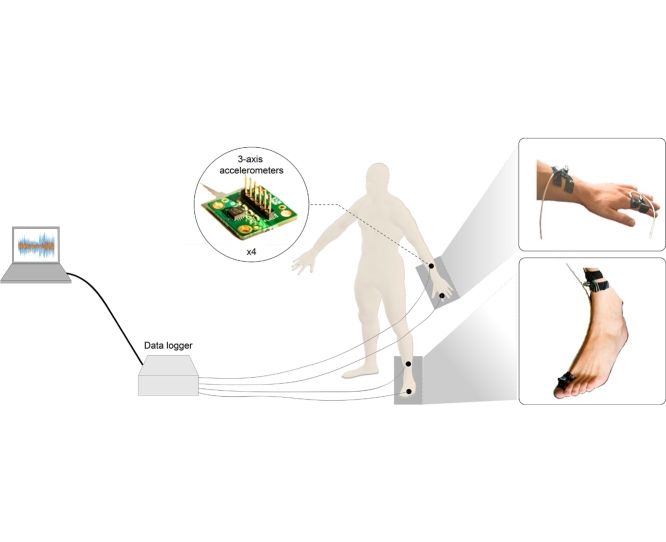

**Table:** 

Subject Area	Medicine and Dentistry
More specific subject area:	*Neurology*
Method name:	Quantitative continuous movement measurement
Name and reference of original method	Goetz, C.G., Tilley, B.C., Shaftman, S.R., Stebbins, G.T., Fahn, S., Martinez-Martin, P., . . . . the Movement Disorder Society UPDRS Revision Task Force. (2008). Movement Disorder Society-Sponsored Revision of the Unified Parkinson’s Disease Rating Scale (MDS- UPDRS): scale presentation and clinimetric testing results. Movement Disorders, 23, 2129–2170
Resource availability	Appendix 1 in the supplemental files for the article. I attach a copy. I submitted this along with the page proofs.

## Method details

### Rationale

Parkinson’s disease, the second most common neurodegenerative disorder [1] and the fourteenth leading cause of death in the United States [2], is caused by the death of dopaminergic neurons that regulate movement in the substantia nigra pars compacta. Approximately one percent of the population of the United States of America over the age of 60 years is afflicted with Parkinson’s disease [1]. The core features of Parkinson’s disease include bradykinesia plus rest tremor or rigidity [3]. Postural instability is another common symptom of Parkinson’s disease [1].

The assessment of Parkinson’s disease and related conditions currently relies on the history of the illness, the clinical interview, the physical examination, and the completion of structured instruments including the Movement Disorder Society-Sponsored Revision of the Unified Parkinson’s Disease Rating Scale (MDS-UPDRS) [3,4]. The MDS-UPDRS [4], a 132-point rating scale that clinicians and researchers use to quantify symptom severity, including rest tremor, postural tremor, action tremor, and postural instability, is the current gold standard for the assessment of Parkinson’s disease for clinical and research purposes. However, there exist multiple drawbacks to the MDS-UPDRS. The reliance on real-time human vision to quantify small differences in motion is a problem of the MDS-UPDRS. The MDS-UPDRS is semi-quantitative by design, however, in addition to significant inter-rater variability, there is inherent subjectivity in administering this tool, which is not blinded in clinical settings.

### Technology to assess Parkinson’s disease

Multiple devices have been developed to assess individuals with Parkinson’s disease. Although a comprehensive review of the utility of technology to obtain quantitative measurements of the symptoms and signs of Parkinson’s disease [[5], [6], [7]], is beyond the scope of this article, identification of the benefits and disadvantages of key representative tools provides the background for the development of the current tool.

More objective measures of tremor and other movements using accelerometry and other technology devices [[8], [9], [10], [11], [12]] have been discussed for decades, but have not yet been implemented routinely to monitor symptom severity as part of diagnostic tools, clinical assessments, or clinical trials. An accelerometer-based device can be used to quantify motion more precisely than the MDS-UDPRS. Accelerometers are small devices that produce a voltage output proportional to acceleration (Table1). The amplitude, speed, and periodicity of movement can be calculated and expressed graphically.

**Table 1:** Table of characteristics of representative accelerometers.

Product	Number	Vendor	Price (USD)	URL
Small, Low Power, 3-Axis ± 3 g Accelerometer	ADXL335BCPZ	Analog Devices, Norwood, MA	3.83	https://shoppingcart.analog.com/
Wearable, wireless sensor	Shimmer3 IMU Unit	Shimmer – North America, Cambridge, MA	495.00	http://www.shimmersensing.com/products/shimmer3-imu-sensor#download-tab
Low-cost triaxial accelerometer	AC115-3D	Connection Technology Center, Inc., Victor, NY	770.00	https://www.ctconline.com/biaxial_triaxial_accelerometers.aspx?prd=AC115
Triaxial capacitive accelerometer	Slam Stick C	Mide Technology, Medford, MA	1000.00	https://www.mide.com/collections/shock-vibration-data-loggers/products/slam-stick-c
Triaxial accelerometer	3023 Series	Dytran Instruments, Inc., Chatsworth, CA	1199.00	https://www.dytran.com/Accelerometers/
Triaxial capacitive accelerometer	Slam Stick X	Mide Technology, Medford, MA	2000.00	https://www.mide.com/collections/shock-vibration-data-loggers/products/slam-stick-x-metal
Triaxial capacitive accelerometer	Slam Stick S	Mide Technology, Medford, MA	3000.00	https://www.mide.com/collections/shock-vibration-data-loggers/products/slam-stick-s

Several approaches have been proposed to utilize accelerometry to assess persons with Parkinson’s disease.

A smart watch device and an analog accelerometer measured tremor peak frequency, peak power, and power of the first four harmonics for patients with Parkinson’s disease and essential tremor. Mean harmonic peak power differentiated the postural tremor of Parkinson’s disease from essential tremor for both modalities [13]. Tasks recorded on a smartphone correlate with some measurements on the MDS-UPDRS [4,14].

Oscillatory movements, both at rest and at posture, were assessed clinically by a movement disorders specialist and by a biaxial accelerometer [15] on upper and lower extremities of people with Parkinson’s disease and controls. There was less variability of rest and postural tremor frequency in the dominant lower limb of people with Parkinson’s disease than in healthy adults. Additionally the frequency of rest tremors was discordant between upper and lower limbs of people with Parkinson’s disease. Accelerometry may detect subtle alterations in movements of people with Parkinson’s disease that cannot be perceived by expert clinicians [16].

A linear accelerometer on the posterior trunk measured postural instability in persons with Parkinson’s disease [17]. Output from a triaxial accelerometer mounted on the waist identified freezing of gait in individuals with Parkinson’s disease [18].

Postural stability has been assessed through the measurement of acceleration from a mobile device attached to the waist [19] and force platform on which the patient stands [20]; the measurements correlated well [21]. This system provides a valuable tool to gauge the extent of postural instability in persons with Parkinson’s disease. Postural instability is not assessed by the proposed system. A wireless motion-sensor unit [22] on the affected index finger of patients with Parkinson’s disease is more sensitive to change than clinical ratings of bradykinesia, hypokinesia, and dysrhythmia [23].

However, sophisticated systems that can safely be used with human participants (Table 1) require budgets that are not available for the general population. Therefore, we sought to develop a safe and efficacious system to assessment key parameters of movement of people with Parkinson’s disease and related conditions using inexpensive items that are readily available (Table 2). We ardently sought to utilize inexpensive, readily available items. We constructed the equipment so that it was approved by Clinical Engineering at Johns Hopkins Hospital for use in human participants. We have not reviewed the myriad of devices that could possibly be constructed similar to our system. Cheaper equipment may be may be dangerous to apply to humans and other animals. A comprehensive review of all possible devices is beyond the scope of this article.

**Table 2:** Table of characteristics of components of the proposed system.

Product	Number	Vendor	Price (USD)	URL
Four small, low power, 3-axis ± 3 g accelerometers	ADXL335BCPZ	Analog Devices, Norwood, MA	3.83 × 4 = 15.32	https://shoppingcart.analog.com/
Four 16-foot-4.5-inch light, shielded cables	Woods 55213143 16/2 low voltage lighting cable, 100-feet	amazon.com	25.22	https://www.amazon.com/Woods-55213143-Voltage-Lighting-100-Feet/dp/B06XKTBM1B
Portable, USB/ethernet data logger system	DI-710 Series	DATAQ Instruments, Inc., Akron, OH	499.00	https://www.dataq.com/products/di-710/
Six-foot high-speed USB revision 2.0 shielded MSL cable	28 A WG/2C + 24AWG/2C (UL) E305668 type CM 75 °C CSA 204790	USBMAX.com / QCUSA INC., Clearwater, FL	1.59	http://www.usbmax.com/USB_Cables.html

Thus, we developed a accelerometry-based method for the acquisition of motion data from patients with a variety of movement disorders and healthy matched controls for signal processing algorithms that can objectively and reliably analyze the collected data to identify pathognomonic motor patterns of patients’ conditions and that can be used to further objectify and more accurately quantify movement disorder ratings scales like the MDS-UPDRS [4]. While wearable devices are available for placement on the wrist and the ankle, they are not suitable for placement on the fingers and the toes, crucial locations to detect the movements of Parkinson’s disease.

### Construction of instrumentation

**Fig. 1:**
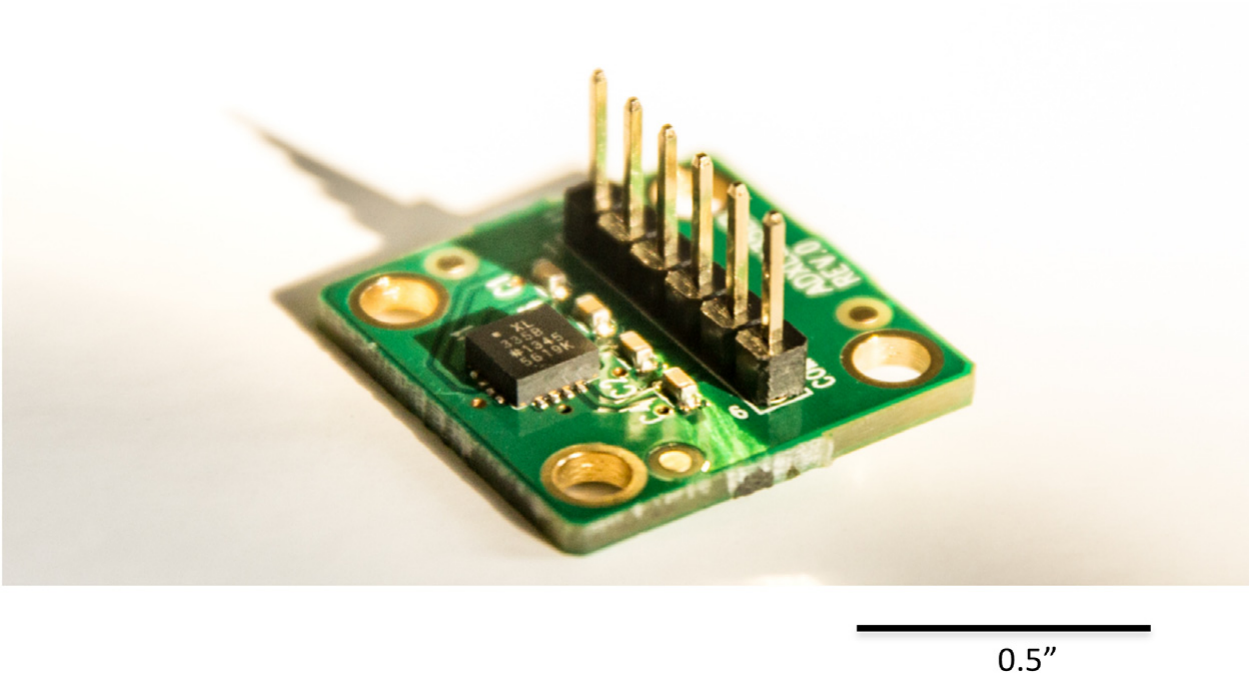
Small, low power, tri-axial ±3 *g* accelerometer [24] mounted on a three-axis accelerometer evaluation board [25].

Each of four small, low-power, tri-axial ±3g accelerometers [24] were individually mounted on a three-axis accelerometer evaluation board [25] (Fig. 1).

*This particular accelerometer was chosen due to its low cost and its ready availability from multiple vendors. Replacements can be obtained quickly. Representative accelerometers are indicated in* Table 1*. The proposed instrumentation has been approved for use with human participants by Clinical Engineering at Johns Hopkins Hospital in Baltimore, Maryland, USA. The safety and efficacy of alternative accelerometers and devices are uncertain. Replacing the accelerometers and devices described in this article by other similarly specified modules may produce unknown effects on the analysis of the data. Before utilizing modules other than those described in this article on human participants, the safety of the devices must be demonstrated*.

**Fig. 2:**
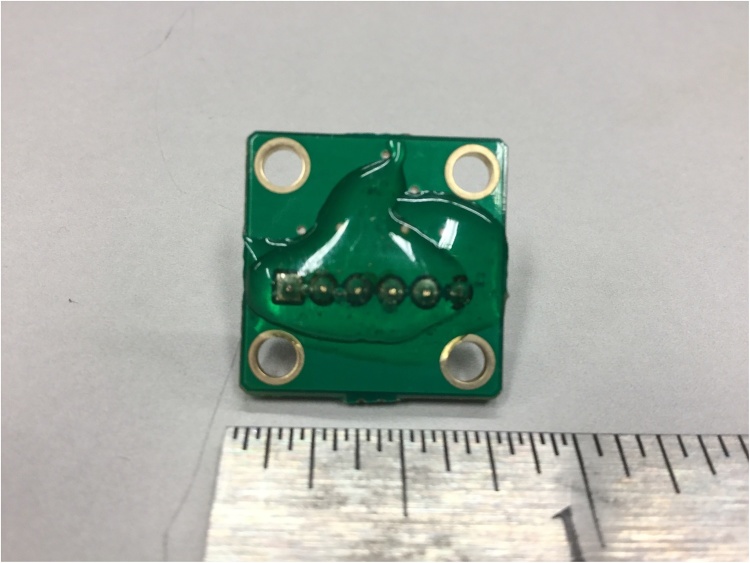
Back surface of a three-axis accelerometer evaluation board (EVAL- ADXL335Z, 2009) with metal pins of accelerometer evaluation board covered with dried epoxy to present a smooth surface when applied to the skin of the participant. When covered with epoxy, the metal pins do not scratch the surface of the skin.

**Fig. 3:**
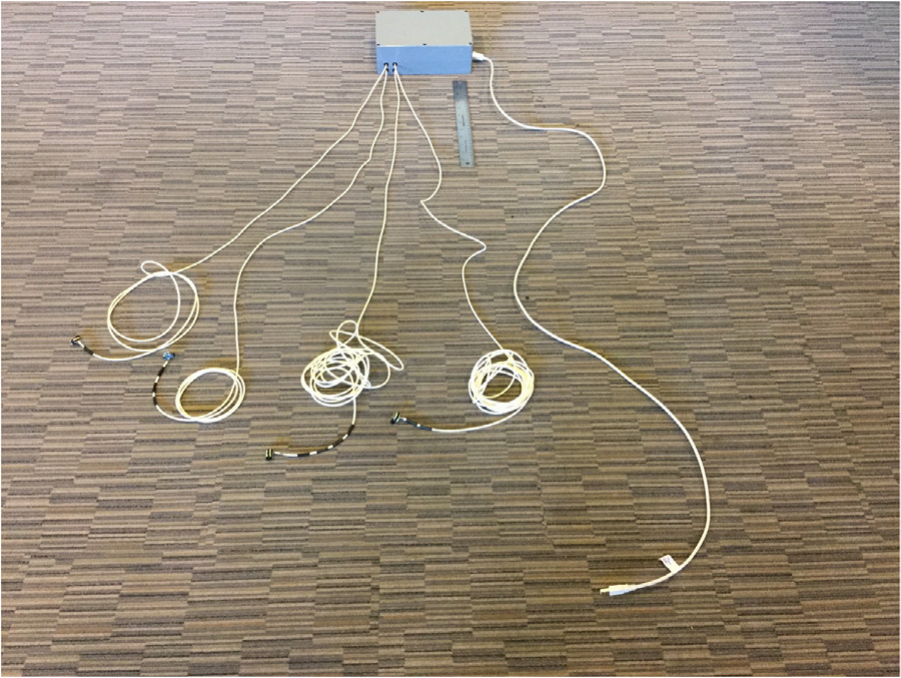
Metal box housing covering low-cost, portable, USB/ethernet data logger system [26] (Fig. 5) with four 16-foot-4.5-inch light, shielded cables identified with one, two, three, or four bands of black tape to connect input from accelerometer evaluation boards board [25] (Fig. 1) on left and a standard 6′ hi-speed USB revision 2.0 shielded 28AWG/ 2C + 24AWG/2C (UL) E305668 type CM 75 °C CSA 204790 type CM 75 °C MSL cable to connect output to a laptop computer on right. A one-foot ruler is displayed by the equipment.

**Fig. 4:**
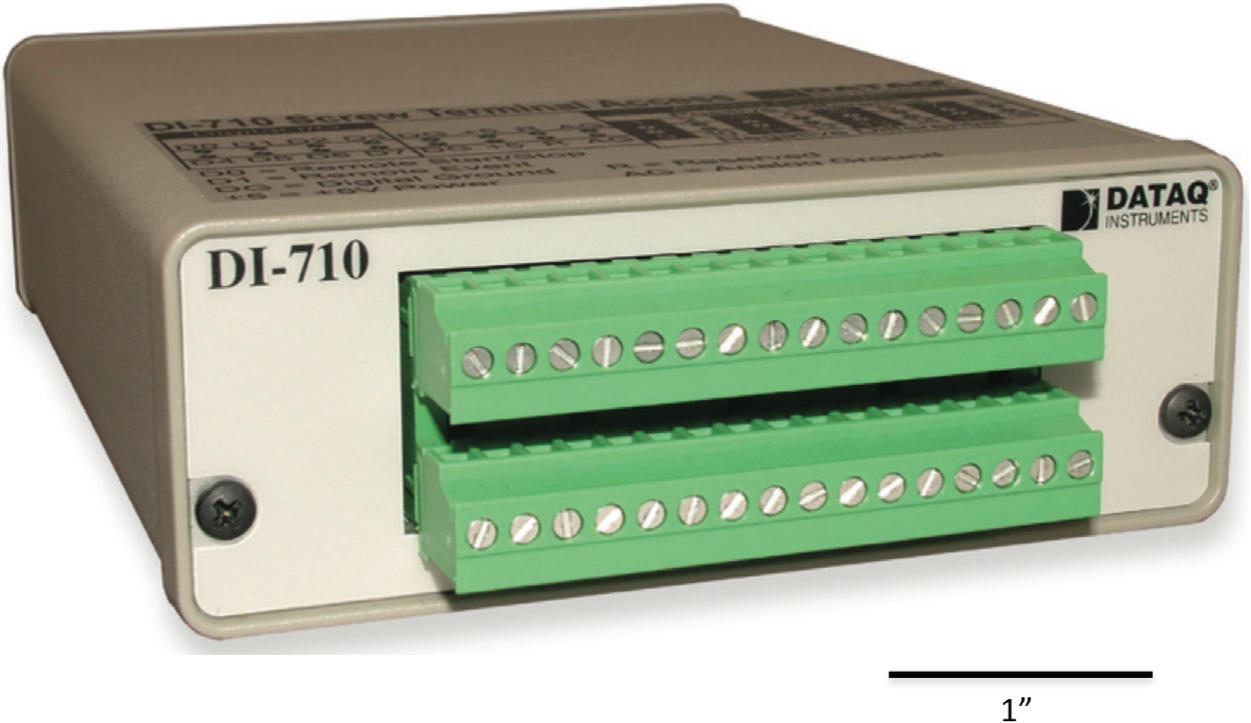
A low-cost, portable, USB/ethernet data logger system (Fig. 5) [26] for accelerometry data acquisition.

**Fig. 5:**
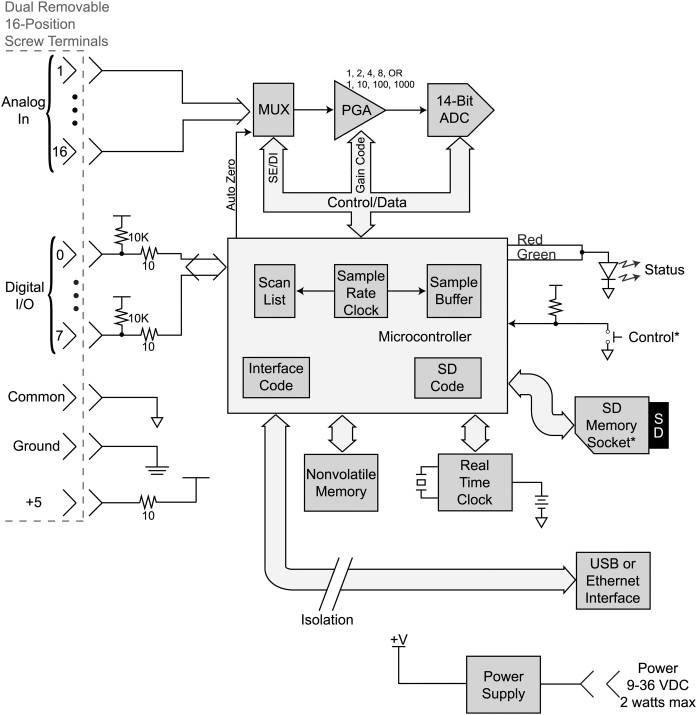
Standard 6′ high-speed USB revision 2.0 shielded 28AWG/2C + 24AWG/2C (UL) E305668 type CM 75 °C CSA 204790 type CM 75 °C MSL cable from the metal box (Fig. 3) being plugged into the laptop computer.

Epoxy was applied to cover the metal pins on the surface of each accelerometer evaluation board to smooth that surface so as to avoid scratching the skin of the patient (Fig. 2). Each accelerometer evaluation board was connected by a 16-foot-4.5-inch light, shielded cable identified with one, two, three, or four bands of black tape (Fig. 3) to a low-cost, portable, USB/ethernet data logger system (Fig. 4) [26] (Fig. 5) [27] secured with soldering inside a metal box of 8.75″ × 5.75″ × 3.00″ that was connected to a laptop computer through a standard 6′ hi-speed USB revision 2.0 shielded 28AWG/2C+24AWG/2C (UL) E305668 type CM 75°C CSA 204790 type CM 75°C MSL cable (Fig. 3). Since the wires may become separated from their connections when the equipment is moved, each wire is firmly soldered in its proper place. The power for the accelerometers is +3 V relative to ground, and the current drawn by the accelerometers is 350 microamps for each accelerometer. The power is supplied by two standard C cell batteries that are housed in a standard battery bracket, and that are physically attached to the data logger [26] with cable ties within the metal box (Fig. 3). No external power is supplied to the data logger [26] or the accelerometer evaluation boards [25] except through the USB cable, which supplies power to operate the data logger [26]. Accelerometry data is acquired using data acquisition and playback software [28] installed on a laptop computer.

Table 2 summarizes the costs of the components of this system. Additionally a laptop computer is utilized for this system. Commercially available systems cost several times [29,30,20] more than the indicated system. Thus, a key advantage of the proposed system is the ready availability of an inexpensive system that can safely be utilized with human participants.

### Recording procedure by trained technologist

**Fig. 6:**
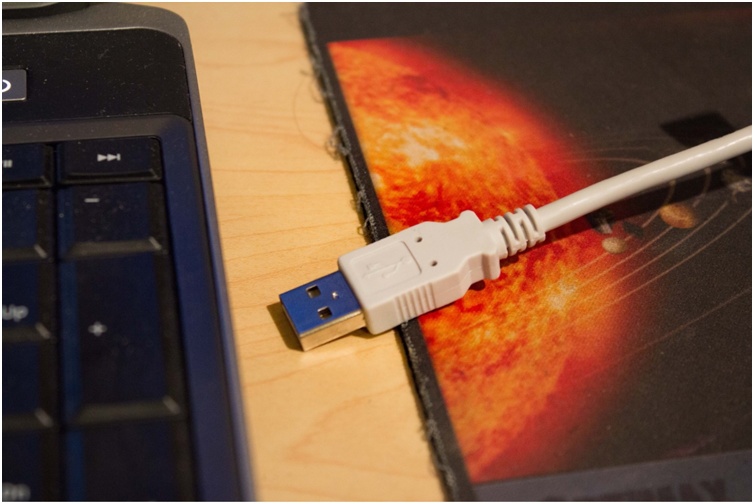
Block diagram of low-cost, portable, USB/ethernet data logger system (Fig. 3, Fig. 4) [27]. Reproduced with permission of Shawn MacDonald, DATAQ Instruments Inc., Akron, Ohio.
• Plug each accelerometer evaluation board (Fig. 1) into the receptacle (Fig. 7) on a 16- foot-4.5-inch light, shielded cable from the metal box (Fig. 3) identified with one, two, three, or four bands of black tape. [Note that the “ST” marking on the accelerometer evaluation board must face towards the side of the red wire. Damage to the accelerometer can result from plugging it in backwards. Also note that the wire number is labelled by strips of black tape (Fig. 3). The wire number must be aligned with placement on the limbs.]

The data from the instrumentation is recorded by a trained technologist who codes and saves on a laptop computer the output of each item. The technologist performed the steps as follows:

• Turn on the laptop computer.
• Plug the standard 6′ hi-speed USB revision 2.0 shielded 28AWG/2C+24AWG/2C (UL) E305668 type CM 75°C CSA 204790 type CM 75°C MSL cable from the metal box (Fig. 3) into the laptop computer (Fig. 6).

**Fig. 7:**
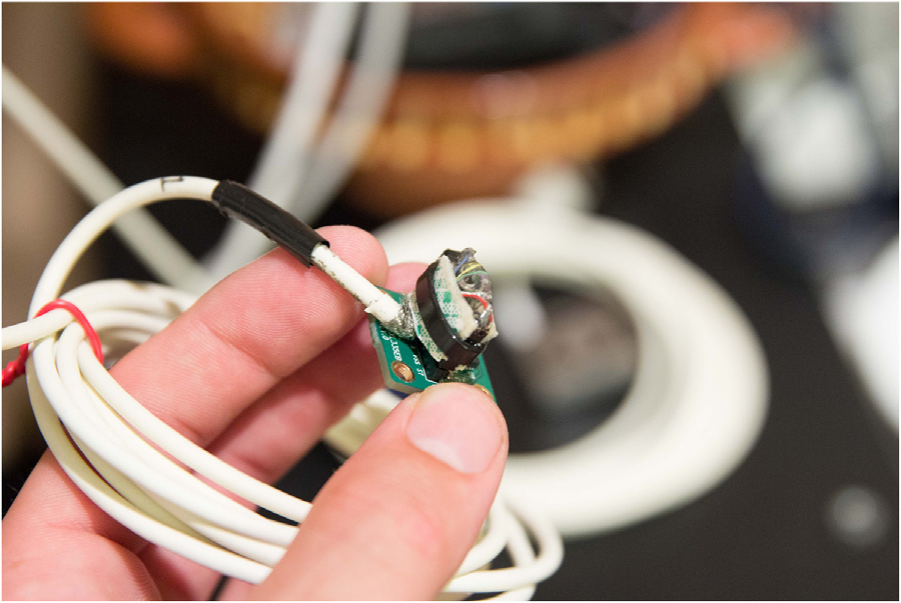
An accelerometer evaluation board [25] plugged into the receptacle on a 16-foot-4.5-inch light, shielded cable from the metal box (Fig. 3).
• With the accelerometer evaluation boards in place on the patient, start the DataQ software [28] by clicking the icon on the desktop (Fig. 8).

**Fig. 8:**
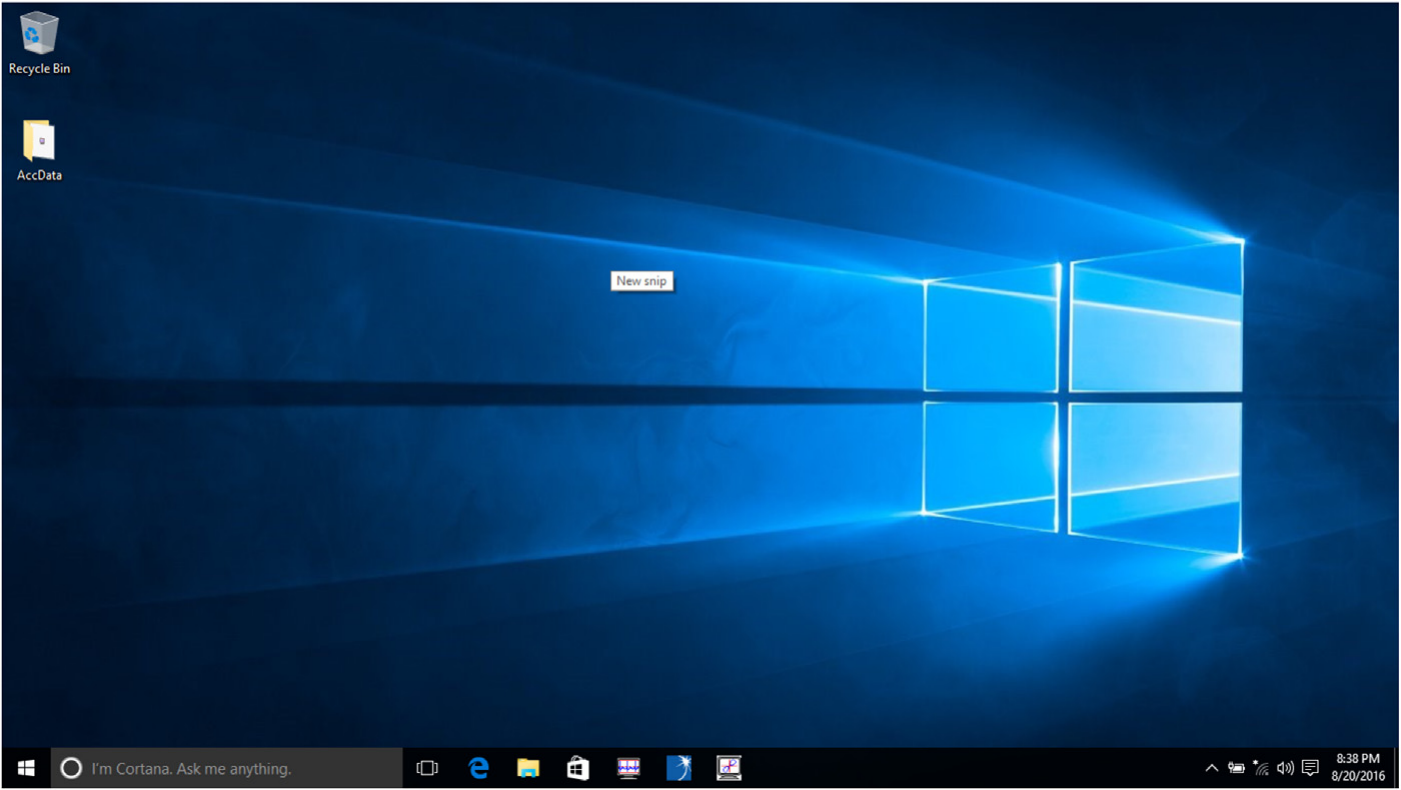
Computer screen with DataQ icon on lower margin.
• When the “DataQ Instruments Hardware Manager” window pops up, click “Start WINDAQ” [28] (Fig. 9). [Twelve thin, dark blue lines running horizontally across the window, each representing an x, y, z component of one of the accelerometers, will appear on the screen (Fig. 10). Note that if any of these lines appears a cyan color, the attachment of the accelerometer is not complete.]

**Fig. 9:**
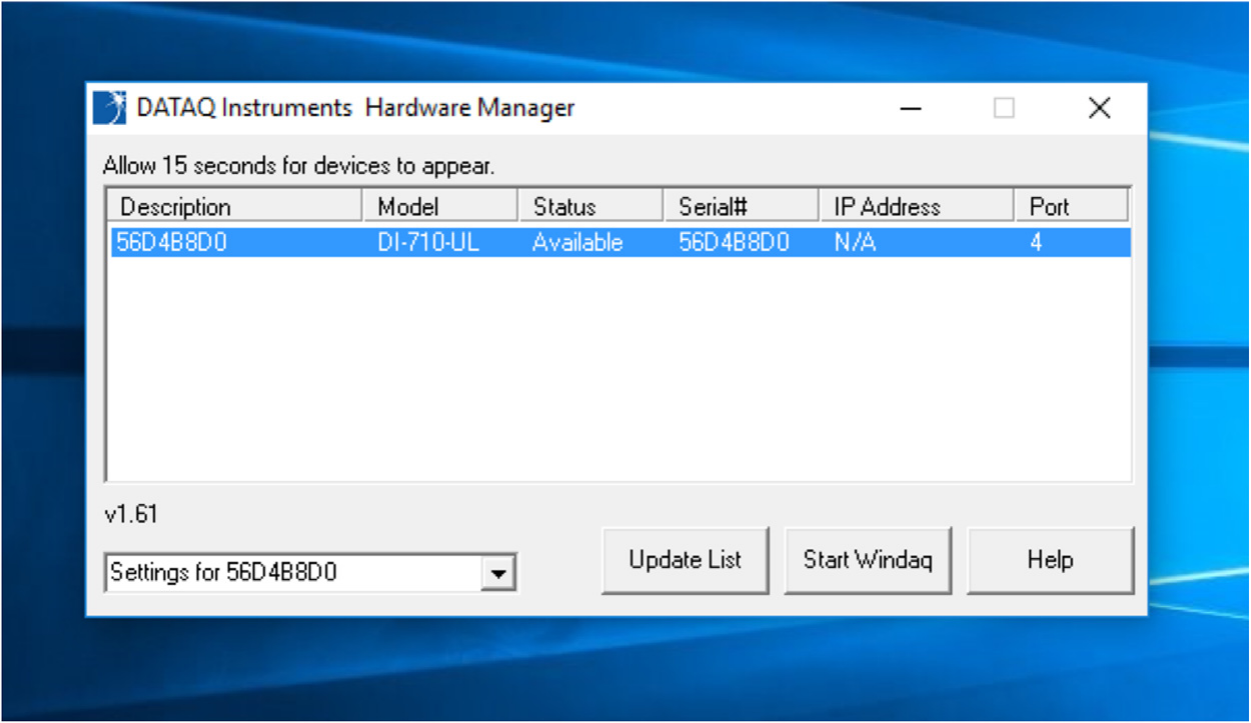
Computer screen after clicking “Start WINDAQ” on the “DataQ Instruments Hardware Manager” window.

**Fig. 10:**
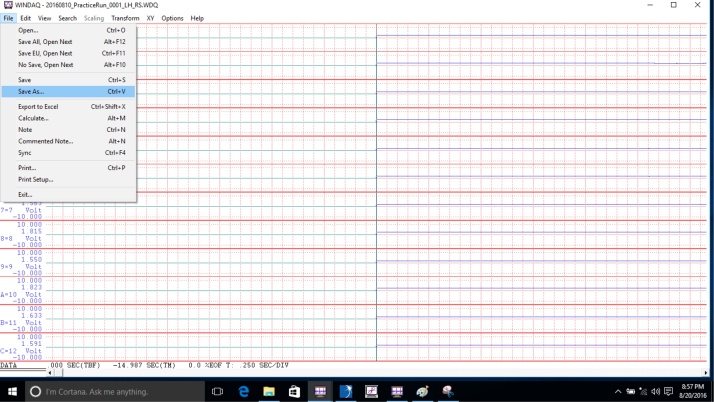
Computer screen displaying twelve thin, dark blue lines running horizontally across the window, each representing an x, y, z component of one of the accelerometers. Note that if any of these lines appears a cyan color, the attachment of the accelerometer evaluation board is incomplete.
• Instruct the examiner to attach the accelerometer evaluation boards to the participant.
• Record data by clicking “File” and then clicking “Record.”
• Choose the file name (Fig. 11).

**Fig. 11:**
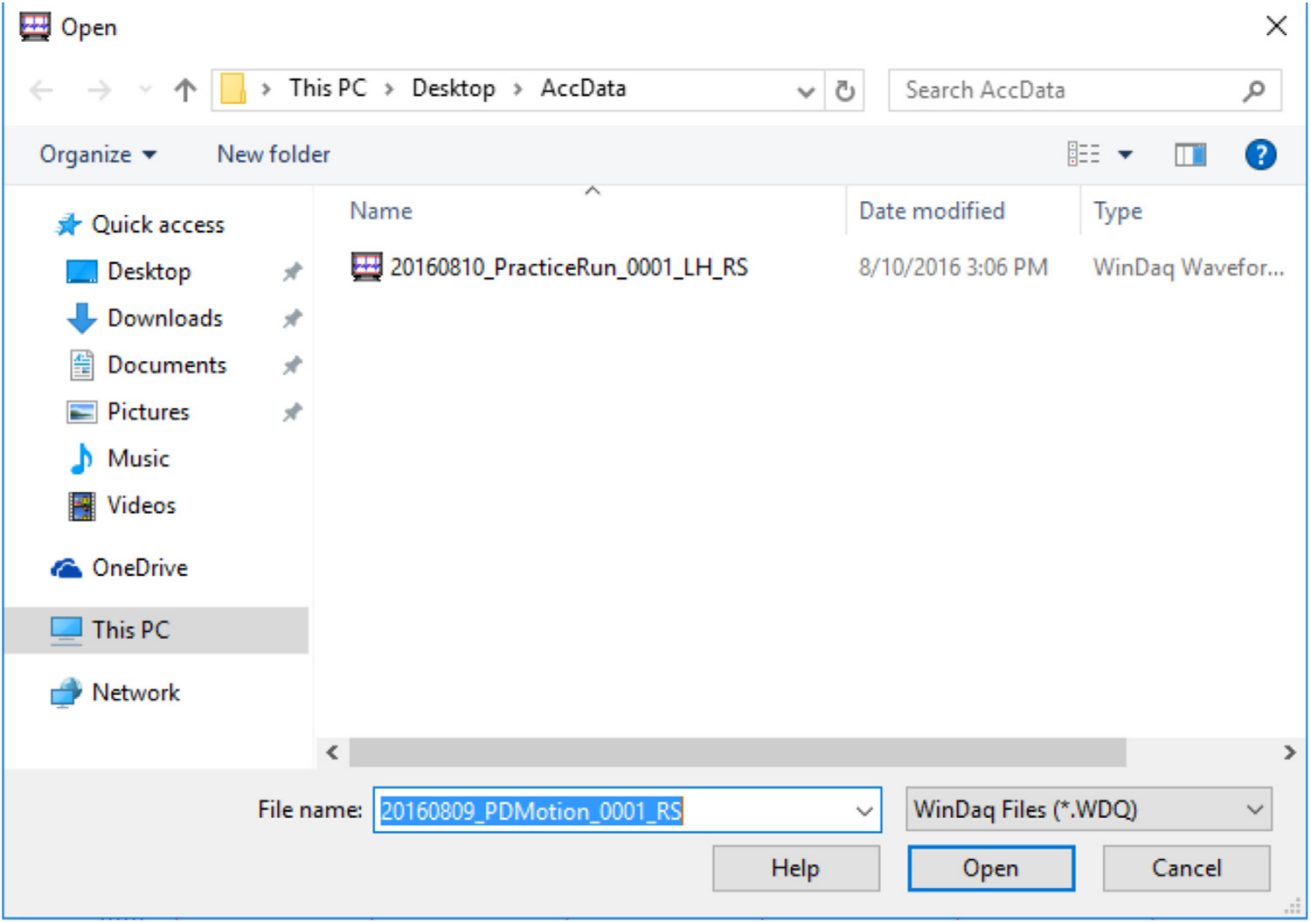
The name of the file for each item of the protocol is entered on the computer.
• Select the duration of the recording time based on the length of time of the item (Fig. 12).

**Fig. 12:**
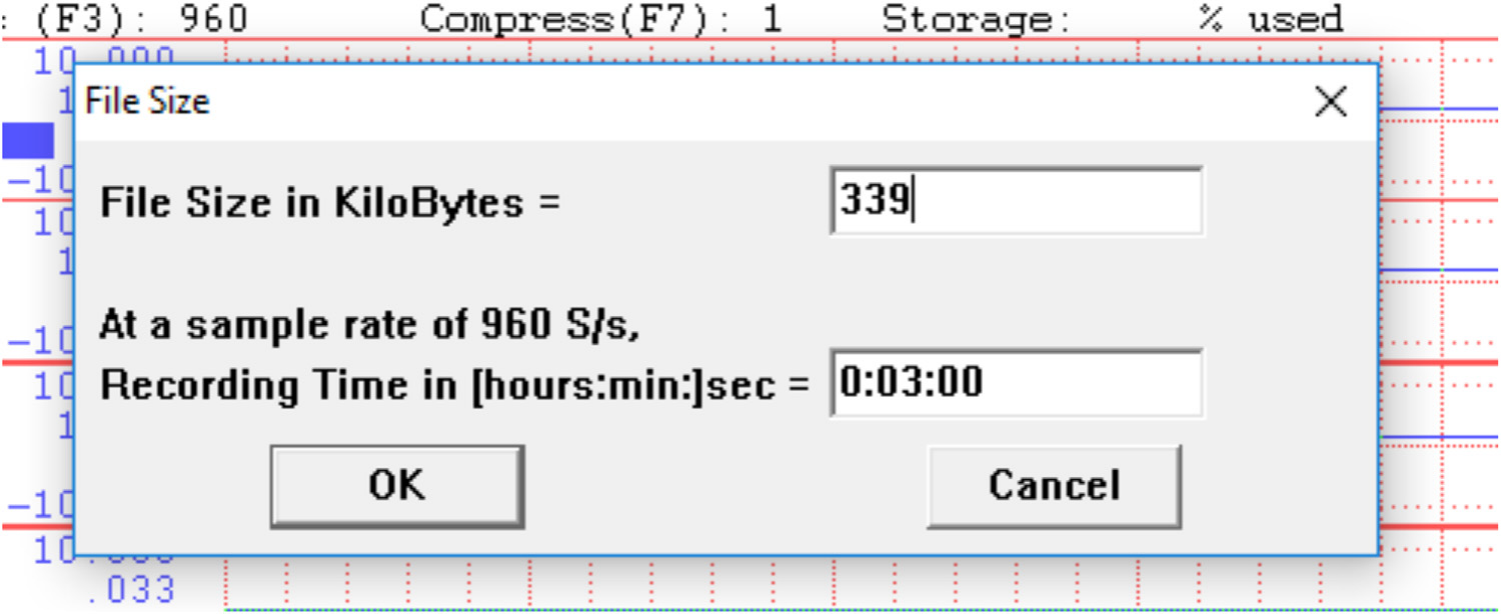
The selection of the duration of the recording of each item is based on the length of time to administer the item.
• When instructed by the examiner, click “OK” so that the device will begin recording. [The file will be saved on the desktop in a folder titled “AccData” (Fig. 13).]

**Fig. 13:**
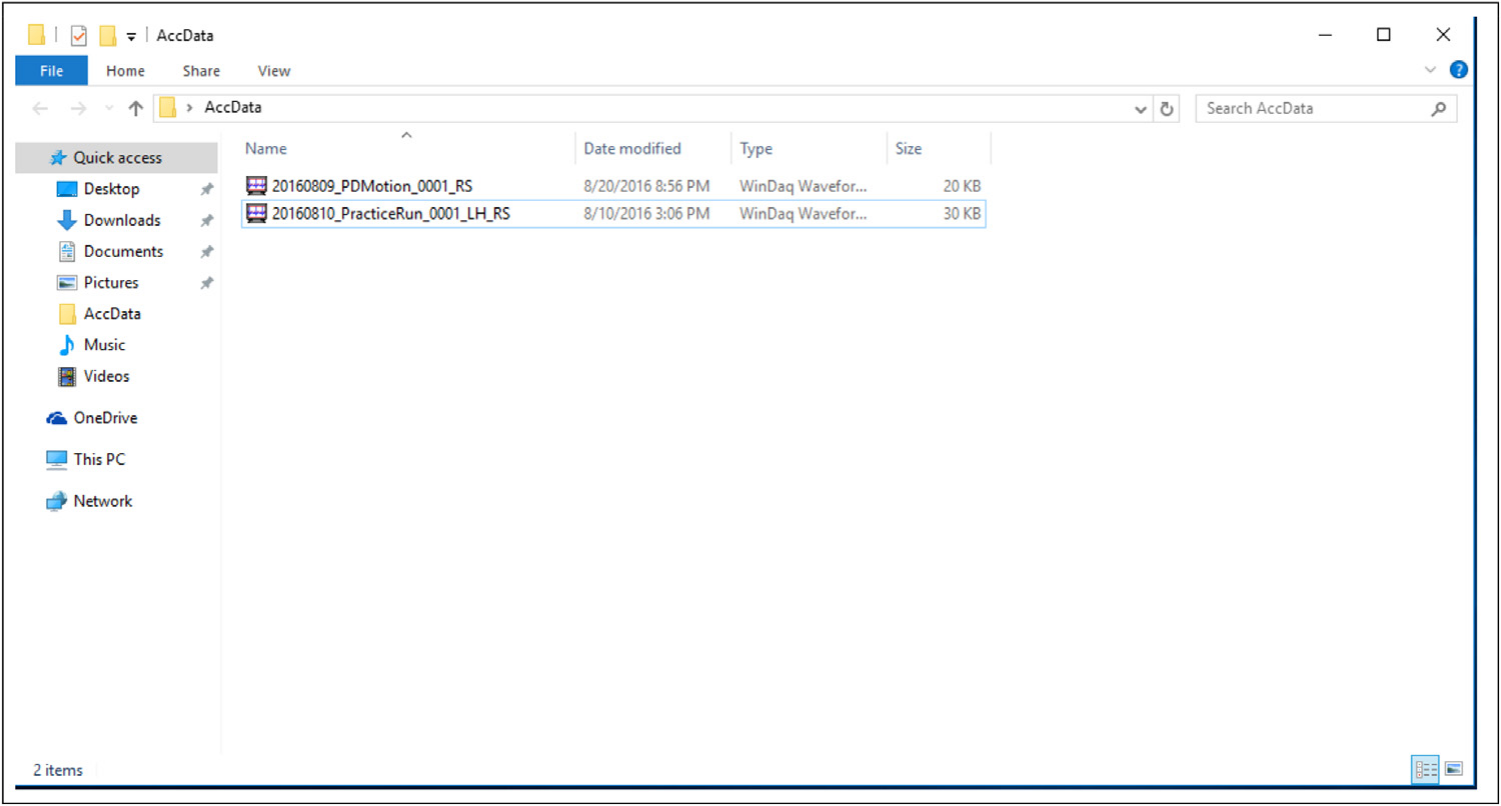
Display of the recorded file from a protocol item in “AccData.”.
• After the participant has completed all the motor tasks, convert the data using the Waveform Browser before analysis.
• Migrate to the desktop, open the folder titled “AccData,” and double click the file to convert. [The file will open in the Waveform Browser.]
• Click “File,” then click “Save As” (Fig. 14).

**Fig. 14:**
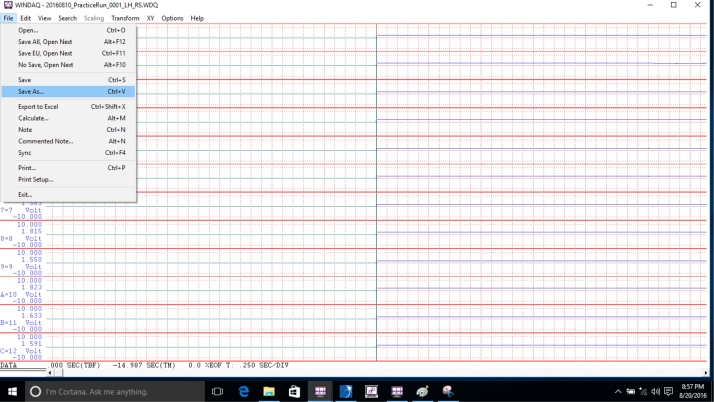
Display to save the data of the recorded file from a protocol item.
• Select the option for ASCII output, then click “Save” (Fig. 15). [Note that these steps must be done for every recording made.]

**Fig. 15:**
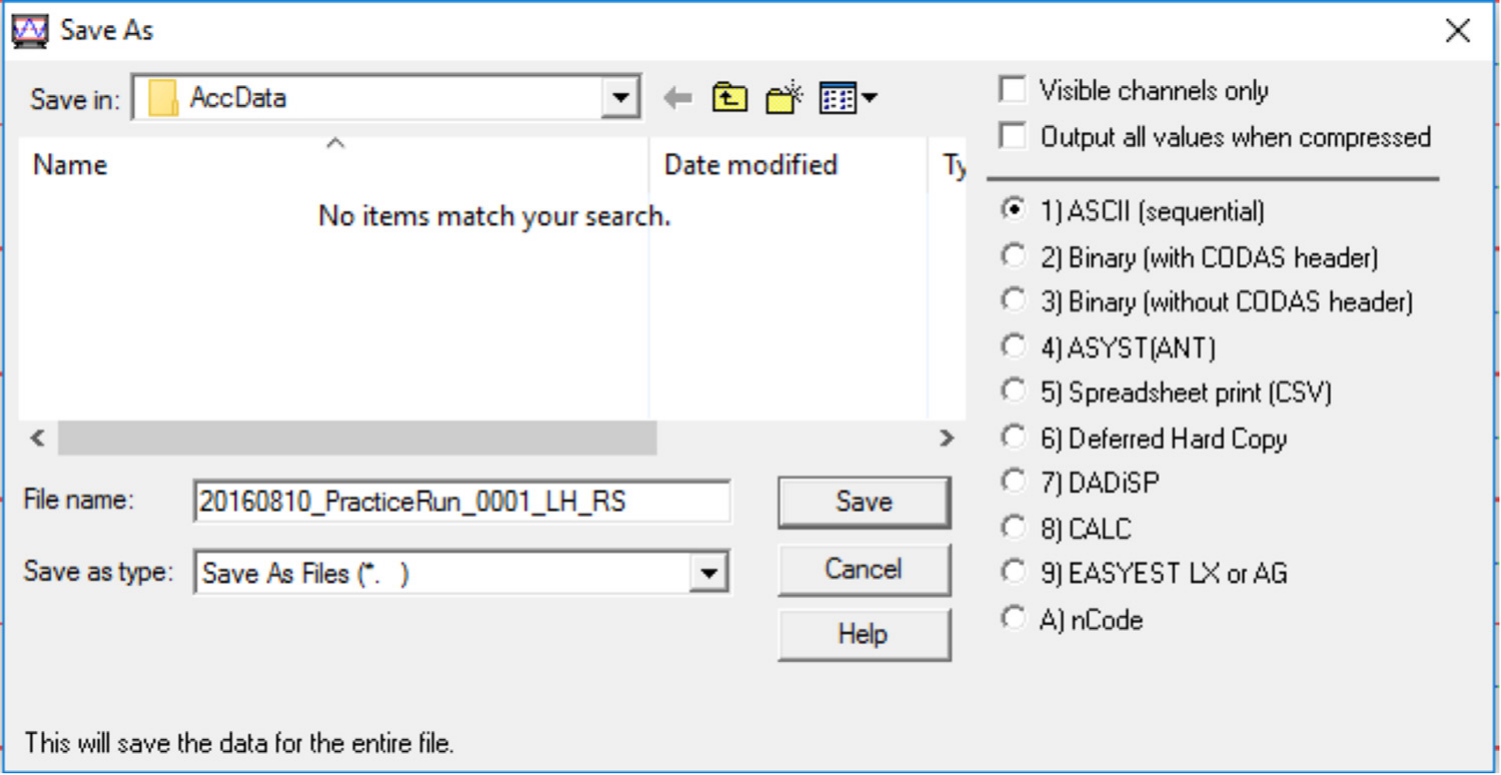
Display to save the data file in the ASCII format.

### Administration of movement assessment to participants with Parkinson’s disease and related conditions

The work was carried out in accordance with the recommendations of the World Medical Association in the WMA Declaration of Helsinki - Ethical Principles for Medical Research Involving Human Subjects [31] and the Recommendations for the Conduct, Reporting, Editing, and Publication of Scholarly Work in Medical Journals [32]. The study was approved by the Institutional Review Board of The Johns Hopkins University School of Medicine in Baltimore, Maryland, United States. All participants provided written informed consent to take part in this study.

The procedure is filmed by a videographer for rating by trained raters certified in the administration of the MDS-UPDRS) [4] by the International Parkinson and Movement Disorder Society (https://mds.movementdisorders.org/updrs/) and blind to the status of the patient. The filmed images are correlated with the output of the instrumentation. Assistants are positioned to catch the patient if there is a fall.

The participant sits on a strong firm chair with a straight back and firm arms placed at least six inches from a wall to facilitate the administration of the procedure. The chair is place far from the wall so that the head of the participant will not touch the wall. Since people with Parkinson’s disease may experience unsteadiness when arising, the chair is placed far enough from the wall so that the participant’s head will not bang against the wall if there is a sudden loss of balance. The examiner and assistants are prepared to catch the participant if the participant falls during the procedure.

The protocol is administered by an experienced examiner who is certified in the administration of the MDS-UPDRS [4] by the International Parkinson and Movement Disorder Society (https://mds.movementdisorders.org/updrs/).

After administering each item the examiner scores each item circling the observed behaviors and recording the side (left or right) of each finding on the coding form (See Appendix 1 in Supplementary materials). The examiner adds notes about other events observed during the administration of each item.

### Administration of the movement assessment of the upper extremities

The participant is queried if there is an allergy to hypoallergic tape. If an allergy is present, then the procedure is aborted.

**Fig. 16:**
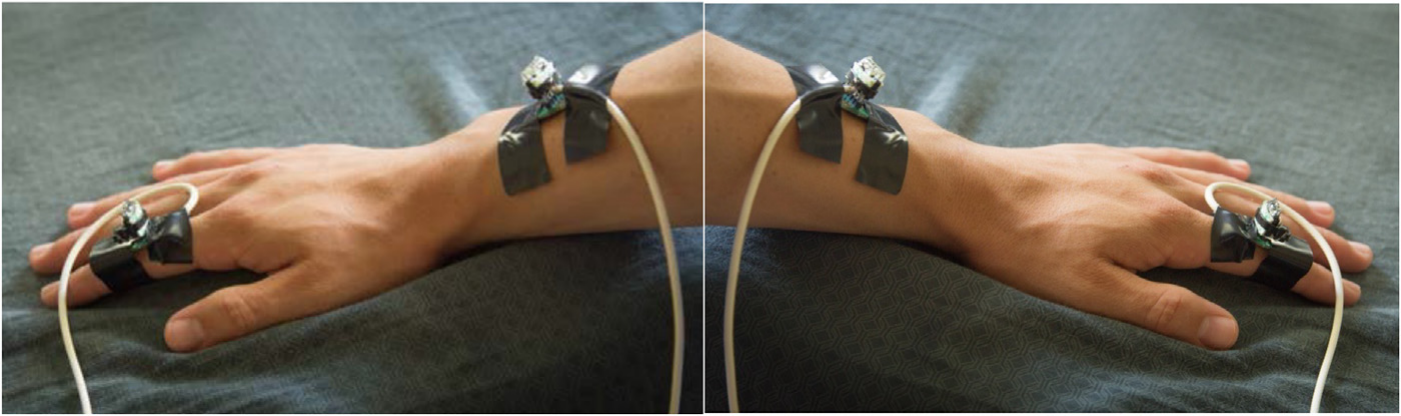
(Left panel) Placement of accelerometer evaluation boards on the dorsal surface of the second (middle) phalanx of the right index finger and midway between radius and ulna approximately two inches proximal to the right wrist joint on the dorsum of the arm. (Right panel) Placement of accelerometer evaluation boards on the dorsal surface of the second (middle) phalanx of the left index finger and midway between radius and ulna approximately two inches proximal to the left wrist joint on the dorsum of the arm.

The accelerometer evaluation boards are attached to the index fingers and wrists as illustrated in Fig. 16 with hypoallergenic tape as follows:

Accelerometer evaluation board 1 – Dorsal surface of the second (middle) phalanx of the right index finger,

Accelerometer evaluation board 2 – Midway between radius and ulna approximately two inches proximal to the right wrist joint on the dorsum of the arm,

Accelerometer evaluation board 3 – Dorsal surface of the second (middle) phalanx of the left index finger,

Accelerometer evaluation board 4 – Midway between radius and ulna approximately two inches proximal to the left wrist joint on the dorsum of the arm.

The video camera is focused on the hands on the arms of the chair.

### “3.17 Rest tremor amplitude” upper limb

The examiner instructs the participant to **“**sit quietly in a chair with the hands placed on the arms of the chair (not the lap) and the feet comfortably supported on the floor” for three minutes. The examiner asks the recording technologist to state when the three minute observation period begins and ends (See Appendix 1 in Supplementary materials).

### “3.17 Rest tremor amplitude” upper limbs counting

The examiner instructs the participant to **“**sit quietly in a chair with the hands placed on the arms of the chair (not the lap) and the feet comfortably supported on the floor” while counting aloud from 30 backwards. The examiner tells (1) the recording technologist to begin recording, (2) the participant to perform the task, and (3) the recording technologist to stop recording (See Appendix 1 in Supplementary materials).

The video camera is focused on the outstretched hands.

### "3.15 Postural tremor of the hands

The examiner advises the recording technologist that one recording will include both right and left procedures. The recording technologist is asked to provide the start and the stop times for the procedure for each hand. The examiner instructs (1) the recording technologist to begin recording, (2) the participant to **“**stretch the right arm out in front of the body with palms down,” the wrist straight, and “the fingers comfortably separated so that they do not touch each other,” (3) the recording technologist to give the start and stop signals for ten seconds, (4) the participant to **“**stretch the left arm out in front of the body with palms down,” the wrist straight, and “the fingers comfortably separated so that they do not touch each other,” (5) the recording technologist to give the start and stop signals for ten seconds, and (6) the recorder to stop recording (See Appendix 1 in Supplementary materials).

### 3.4 Finger tapping

The examiner advises the recording technologist that one recording will include both right and left procedures. The examiner demonstrates the task, but does not continue to perform the task while the participant is being tested. The examiner instructs (1) the recording technologist to begin recording, (2) the participant to tap the right index finger on the right thumb “as quickly AND as big as possible,” (3) the participant to tap the left index finger on the left thumb “as quickly AND as big as possible, and 4) the recording technologist to stop recording (See Appendix 1 in Supplementary materials).

### 3.5 Hand movements

The examiner advises the recording technologist that one recording will include both right and left procedures. The examiner demonstrates the task, but does not continue to perform the task while the participant is being tested. The examiner instructs (1) the recording technologist to begin recording, (2) the participant to make a tight fist with the right “arm bent at the elbow so that the palm faces the examiner,” and then “to open the hand as fully AND as quickly as possible,” (3)) the participant to make a tight fist with the left “arm bent at the elbow so that the palm faces the examiner,” and then “to open the hand as fully AND as quickly as possible,” and 4) the recording technologist to stop recording (See Appendix 1 in Supplementary materials).

### 3.6 Pronation-supination movements of hands

The examiner advises the recording technologist that one recording will include both right and left procedures. The examiner demonstrates the task, but does not continue to perform the task while the participant is being tested. The examiner instructs (1) the recording technologist to begin recording, (2) the participant to “extend the right arm out in front of his/her body with the palm down; then to turn the palm up and down alternately as fast and as fully as possible,” (3) the participant to “extend the left arm out in front of his/her body with the palm down; then to turn the palm up and down alternately as fast and as fully as possible,” and 4) the recording technologist to stop recording (See Appendix 1 in Supplementary materials). The video camera is focused to capture the full body from head to toe while standing.

### “3.9 Arising from chair” upper limbs

The examiner asks the recording technologist to begin recording. The examiner demonstrates the task, but does not continue to perform the task while the participant is being tested. The examiner asks the participant to “sit in a straight-backed chair with arms, with both feet on the floor and sitting back in the chair (if the patient is not too short),” with arms crossed across the chest and then to stand up. The examiner asks the recording technologist to stop recording (See Appendix 1 in Supplementary materials). The accelerometer evaluation boards are removed from the fingers and wrists.

### Administration of the movement assessment of the lower extremities

The participant is asked to remove shoes and socks.

**Fig. 17:**
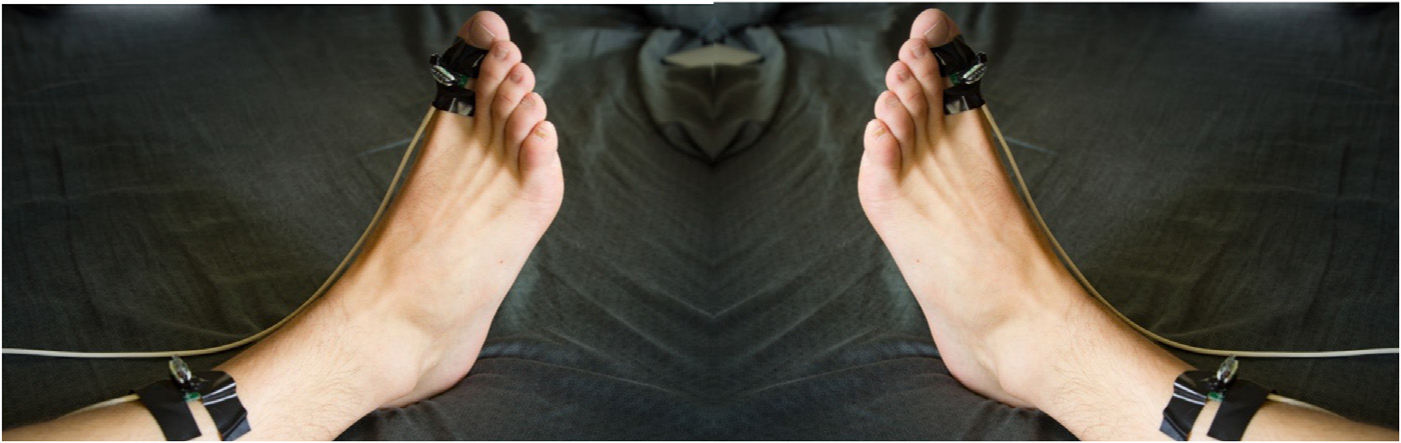
(Left panel) Placement of accelerometer evaluation boards on the anterior surface of the right tibia two inches proximal to the medial malleolus and on the dorsal surface of the proximal phalanx of the right big toe. (Right panel) Placement of accelerometer evaluation boards on the anterior surface of the left tibia two inches proximal to the medial malleolus and on the dorsal surface of the proximal phalanx of the left big toe.

The accelerometer evaluation boards are attached to the shins and toes as illustrated in Fig. 17 with hypoallergenic tape as follows:

Accelerometer evaluation board 1 – Anterior surface of the right tibia two inches proximal to the medial malleolus.

Accelerometer evaluation board 2 – Dorsal surface of the proximal phalanx of the right big toe.

Accelerometer evaluation board 3 – Anterior surface of the left tibia two inches proximal to the medial malleolus.

Accelerometer evaluation board 4 – Dorsal surface of the proximal phalanx of the left big toe.

### “3.9 Arising from chair” lower limbs

The examiner asks the recording technologist to begin recording. The examiner demonstrates the task, but does not continue to perform the task while the participant is being tested. The examiner asks the participant to “sit in a straight-backed chair with arms, with both feet on the floor and sitting back in the chair (if the patient is not too short),” with arms crossed across the chest and then to stand up. The examiner asks the recording technologist to stop recording (See Appendix 1 in Supplementary materials). The video camera is focused on the feet on the floor.

### “3.17 Rest tremor amplitude” lower limbs

The examiner instructs the participant to **“**sit quietly in a chair with the hands placed on the arms of the chair (not the lap) and the feet comfortably supported on the floor” for three minutes. The examiner asks the recording technologist to state when the three minute observation period begins and ends (See Appendix 1 in Supplementary materials).

### “3.17 Rest tremor amplitude” lower limbs counting

The examiner instructs the participant to sit quietly in a chair with the hands placed on the arms of the chair (not the lap) and the feet comfortably supported on the floor” while counting aloud from 30 backwards. The examiner tells (1) the recording technologist to begin recording, (2) the participant to perform the task, and (3) the recording technologist to stop recording (See Appendix 1 in Supplementary materials).

The video camera is focused on the feet in motion.

### 3.7 Toe tapping

The examiner advises the recording technologist that one recording will include both right and left procedures. The examiner demonstrates the task, but does not continue to perform the task while the participant is being tested. The examiner instructs (1) the recording technologist to begin recording, (2) the participant to place the right “heel on the ground in a comfortable position and then tap the toes as big and as fast as possible,” (3) the participant to place the left “heel on the ground in a comfortable position and then tap the toes as big and as fast as possible,” and (4) the recording technologist to stop recording (See Appendix 1 in Supplementary materials).

### 3.8 Leg agility

The examiner advises the recording technologist that one recording will include both right and left procedures. The examiner demonstrates the task, but does not continue to perform the task while the participant is being tested. The examiner instructs (1) the recording technologist to begin recording, (2) the participant to place the right “foot on the ground in a comfortable position and then raise and stomp the foot on the ground as high and as fast as possible,” (3) the participant to place the left “foot on the ground in a comfortable position and then raise and stomp the foot on the ground as high and as fast as possible,” and (4) the recording technologist to stop recording (See Appendix 1 in Supplementary materials).

Portions of the Movement Disorder Society-Sponsored Revision of the Unified Parkinson’s Disease Rating Scale (MDS-UPDRS) [4] in quotation marks are used with the kind permission of the International Parkinson and Movement Disorder Society.

## Method validation

**Fig. 18:**
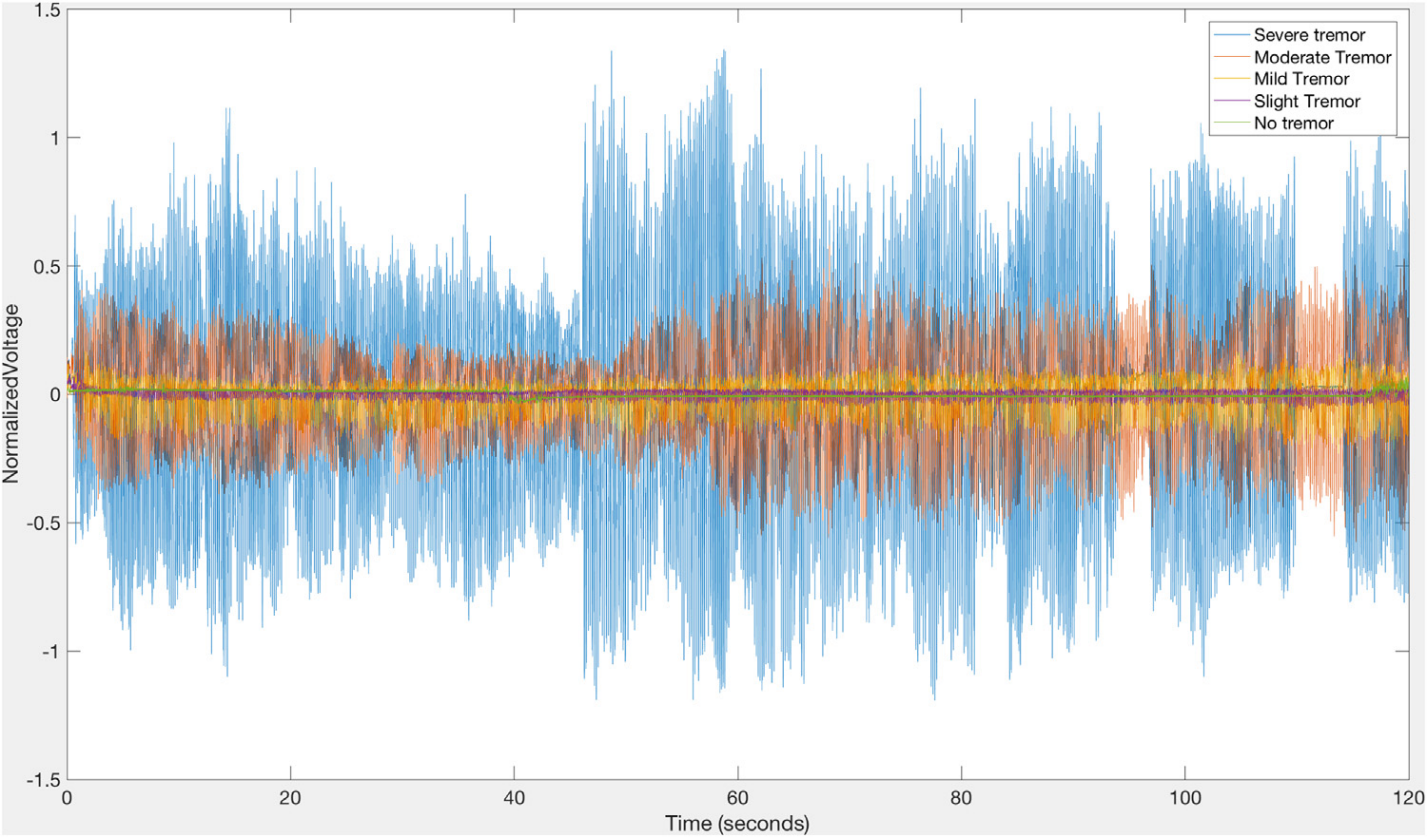
Representation of a tremor imitation by a healthy 25-year-old man without Parkinson’s disease. The ordinate represents the normalized voltage output of the device versus time on the abscissa. Tremor acceleration amplitude increases with increasing tremor severity.

**Fig. 19:**
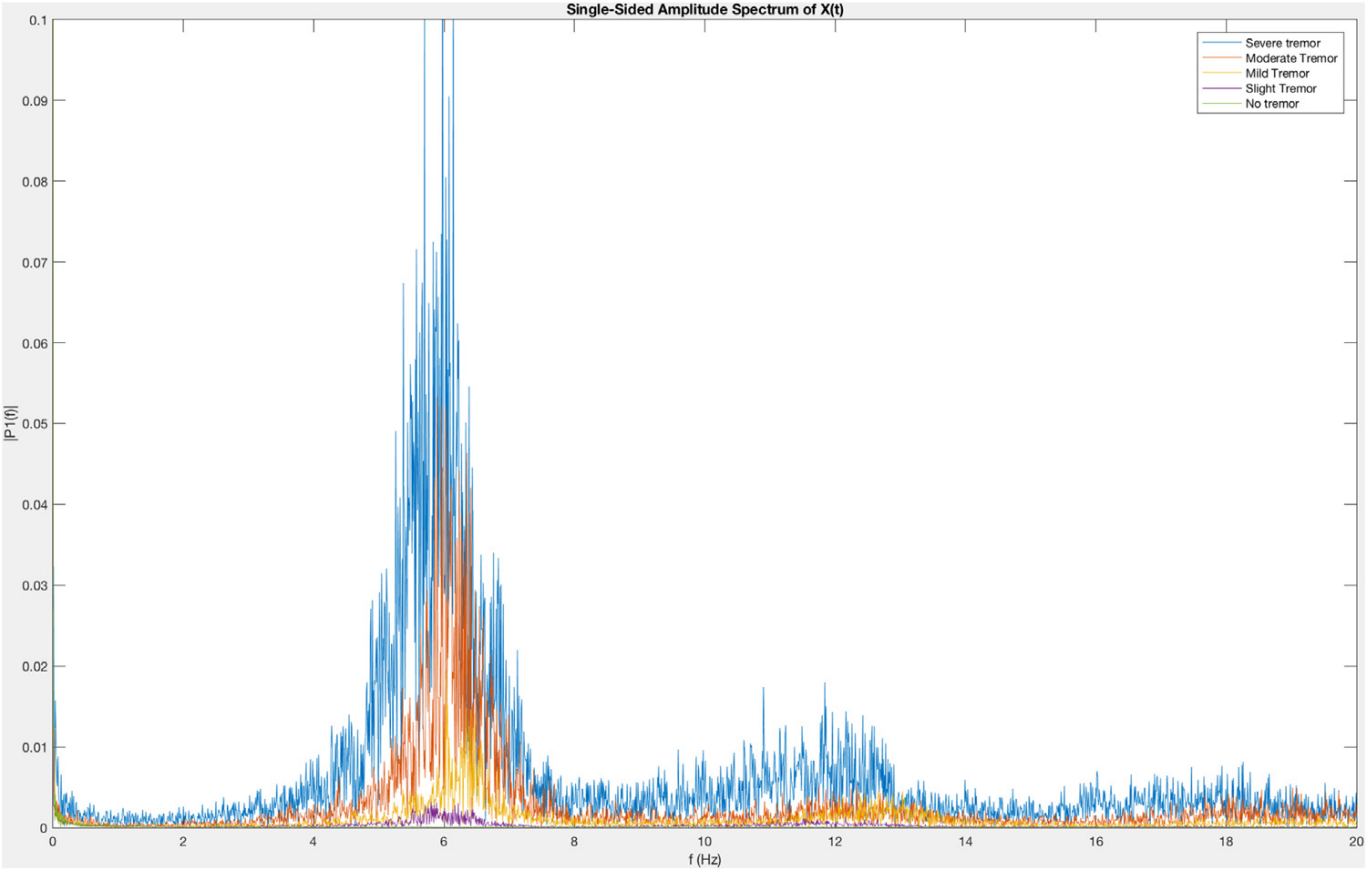
Representation of a tremor imitation by a healthy 25-year-old man without Parkinson’s disease. Sample signal processing algorithm by means of a fast Fourier transform (FFT) plot of the same data of Fig. 18, where the ordinate represents the single-sided amplitude corresponding to the abscissa frequency.

The method was applied to the hand of a healthy 25-year-old man without Parkinson’s disease who generated recording without a tremor and with imitated tremors of slight, mild, moderate, and severe magnitude. Normal participants are often able to simulate tremors that are indistinguishable from those of patients with Parkinson’s disease [33]. Signal processing algorithms [34] were written to extract and analyze the data. Fig. 18 demonstrates the representation of the data of a healthy 25-year-old man without Parkinson’s disease mimicking tremors rated as “no tremor,” “slight tremor,” “mild tremor,” “moderate tremor,” and “severe tremor” according to the MDS-UPDRS [4]. Tremor acceleration amplitude increases with increasing tremor severity (Fig. 18). Fast Fourier transforms (FFTs) [[35], [36], [37]] of the data of Fig. 18 demonstrate that for all degrees of tremor severity, there is a fundamental rhythm at 6 Hz with harmonics at 12 Hz and 8 HZ (Fig. 19). Additional analyses of data are being developed [38,39].

The protocol has been performed on 20 participants with Parkinson’s disease and one participant with multiple system atrophy (MSA), a clinical disorder with some features of Parkinson’s disease [40], and three age-matched healthy control participants. Test-retest has been performed on eight participants with Parkinson’s disease [41] and three healthy control participants. These data are being analyzed for future publications.

## Limitations

The proposed system does not measure action tremor and postural instability, key aspects of the symptomatology of Parkinson’s disease. During some of the initial practice sessions the wires became separated from the accelerometer due to vigorous movements. Therefore, we did not include the measurement of action tremor for fear that the wires would be dislodged. By firmly attaching the equipment with solder and epoxy, we are likely to construct secure attachments that can withstand the force of actions movements. We shall include assessment for action tremor in future studies. We also avoided specific tests of postural stability for fear of disconnecting the wires during the process. By making strong connections of the wires to the equipment, we shall include a balance board to measure the center of pressure during standing to test postural instability in future studies.

## Future directions

The proposed instrumentation offers a simple, relatively inexpensively constructed approach to generate quantitative continuous measurement of movements in the extremities of people with Parkinson's disease. There are several methods to analyze the data obtained currently. Additionally, there are several methods to enhance the current protocol to capture the spectrum of movement abnormalities characteristic of Parkinson’s disease to provide a comprehensive representation of the common deficits manifested by persons with Parkinson’s disease. Therefore, we shall describe signal processing approaches to characterize the motor symptoms of Parkinson’s disease by means of quantification algorithms for the current instrumentation and for enhanced instrumentation.

### Quantification algorithms for the current instrumentation

Currently we measure the specified movements in the upper and lower extremities by means of visual observation scored during the live rating and by subsequent videotape review along with analysis of the output of the instrumentation. Analysis of the output of the current instrumentation includes the application of fast Fourier transforms to represent the amplitude of the normalized voltage of tremors as a function of time (Fig. 18) [38,40,39,41,35]. This approach is being applied to the output of cohorts of persons with Parkinson’s disease and age-matched healthy controls [38,40,39,41,35]. The analysis of the output of instrumentation can be enhanced by the application of time-frequency analysis with the bump variant of the continuous wavelet transform [42,37,38]. The output gives a visual representation of both the amplitude of the normalized voltage and the frequency as functions of time. With this approach the viewer may detect increasing and decreasing frequencies as well as halts and interruptions of movements ([42,37]; Harrigan et al., 2018). Additionally application of machine learning [43,36] is a promising approach for the analysis of the current protocol. For example, downsampling strategies [44] may be applied to the output of the instrumentation to generate quantification algorithms to characterize the motor symptoms of Parkinson’s disease.

### Quantification algorithms for future enhanced instrumentation

We plan to enhance the current protocol for the quantification of the motor symptoms of Parkinson’s disease with the addition of wearable instrumentation, including accelerometers, compasses, electromyograms, and gyroscopes, and other hardware, such as force plates, to capture aspects of movements including halts and balance problems. Additionally, systems have been developed to combine wearable instrumentation and stationary platforms with sophisticated software and other analysis procedures. While a comprehensive review of available equipment and the tools to analyze the output is beyond the scope of this article, we would like to suggest some promising approaches. Inertial sensors are often constructed as wearable devices [45].

#### Inertial sensors

##### Accelerometers

Linear acceleration data measures the amplitude of oscillations by means of accelerometers [45,46]. The current method utilizes single accelerometers placed, first on both pointer fingers and both forearm and, second, on both great toes and both shins. Future developments may include placing accelerometers on both pointer and both little fingers, both forearms, both great and both little toes, and both shins at the same time. Thus, assessments of upper and lower extremities may be performed simultaneously. The presence of accelerometers on the medial and lateral aspects of the hands and the feet will facilitate the detection alternating motions in those parts. The output of accelerometers can be analyzed by fast Fourier transforms [35,[38], [39], [40], [41]], continuous wavelet transforms [42] and machine learning [44,36]. A spectrum of accelerometers have been developed [7]. Additional methods of analysis include parametric [47] and non-parametric procedures [16], short time Fourier transforms, power spectral density analysis, peak detection nethods, and pattern recognition [48]. Gravitational artifacts may occur with a vertical hand when the device is mounted near the wrist [49]. Mean harmonic power differentiated the postural tremor of Parkinson’s disease from essential tremor [13].

##### Compasses

Compass sensors have been utilized to provide absolute orientation for telemonitoring [50].

##### Electromyograms (EMGs)

Muscular contractions are measured by means of electromyograms (EMGs), electrode sensors fixed to the skin [45,46,51].

##### Gyroscopes

Gyroscopic sensors are devices to detect angular rotation and measure angular velocity [45,46]. They can be mounted on hands and other body parts. Gyroscopes can identify gait, sway, tremor, and bradykinesia [5]. Fast Fourier transforms readily analyze the outputs of gyroscopes [33]. Output of accelerometers and gyroscopes together are analyzed by means of fast Fourier transforms [43].

##### Systems combining inertial sensor techniques

Some systems combine accelerometers and gyroscopes [5].

#### Force sensors

##### Gait analysis

Gait measures can be made using a motion capture system consisting of reflective microbeads and EMGs attached to the body parts of the participant and recorded by a special video camera for analysis with specialized software [51]. OpenSim Software [52] utilizes measures of acceleration to calculate force. This measurement system reproduces the common measurements on Parkinson’s disease, such as stride lengths and timing of stance phase, and it detects movement artifacts within the ankles, hips, and knees, so that halting of gait, slowing, and tremor can be identified. The standard gait analysis tools for OpenSim calculate joint motion, forces, and moments over time using an inverse kinematics calculation, specialized for each participant. The measurement system also includes surface EMG data acquisition, which detects muscle activation. The EMG measurements indicate halting, slowing, and tremor. The components of a motion capture system are wearable.

##### Ground platforms [5]

A detailed analysis of balance can be made by means of a force plate [20]. The variations in the center of pressure during standing can be measured to test postural instability [53] to identify freezing of gait in Parkinson’s disease [54]. The specific changes in neurological performance that are likely to affect gait and balance can be characterized by tremor, slowing, and halting. Based on biomechanical considerations of motion and posture control [55], the control systems that maintain balance and posture can be considered as feedback control loops. A neurologically induced tremor can be considered a noise input to the systems that control posture and slowing or halting can be considered delays in the feedback and actuation components. Delays in the components of a control system can induce instabilities in mechanical systems [56]. The association between delays and stability in mechanical systems can be reflected in the connection between symptoms of halting and slowing and the postural stability in the measurements in this study.

## Conclusion

A low-cost method to provide quantitative continuous measurement of movements in the extremities of people with Parkinson's disease and correlation with live and video ratings of the movements with modifications of the current state-of-the-art rating scale [4] was presented. Validation of the method with the data from tremors of varying magnitudes simulated by a healthy 25-year-old man without Parkinson’s disease was described. The procedure has also been conducted on 20 people with Parkinson’s disease and one person with multiple system atrophy [40] and three healthy control participants. Test-retest of the procedure has been accomplished on eight of the people with Parkinson’s disease [41] and three healthy control participants. Other methods of data analysis are being developed [38,39]. This procedure will provide the basis to stratify people with Parkinson’s disease and related conditions by patient diagnosis, disease severity, and medication status and to analyze the output with custom signal processing techniques with the goal of identifying pathognomonic patterns of movement distinguishing among different movement disorders. For example, progressive supranuclear palsy may be differentiated from Parkinson’s disease by finger tapping: there is a decrement in amplitude/speed in Parkinson’s disease, whereas in progressive supranuclear palsy there is relative preservation of speed and notable loss of tapping amplitude (hypokinesia). Additionally the proposed procedure will likely facilitate the presence of essential tremor at 8–12 Hz and enhanced physiological tremor at 12–18 Hz. This procedure will provide the basis for construction of comparable equipment in regions without expert clinicians. Application of this procedure by examiners, recording technologists, and videographers trained in the protocol will provide the means to generate electronic output for interpretation by experts. Thus, the protocol will provide the means for patients in disadvantaged regions to benefit from the advice of experts for diagnostic and therapeutic purposes. Additionally the proposed protocol will provide quantitative output to generate biomarkers for specific clinical conditions. The protocol will provide the bases for objective measurements of the progress of participants in clinical trials and other treatment regimens.

## Appendix A Supplementary data

Appendix 1 is a coding form to record the scores of a trained examiner for the patient’s performance of each item of the procedure. The specific items demonstrated by the patient can be encircled on the form along with the note if the finding was observed on the left or the right. The examiner may add any remarks to assist in the interpretation of the findings.

## References

[1] de Lau L.M.L., Breteler M.M.B. (2006). Epidemiology of Parkinson’s disease. Lancet Neurol..

[2] National Parkinson Foundation (2017). What is Parkinson’s?. http://www.parkinson.org/parkinson-s-disease/pd-101/what-is-parkinson-s-disease.

[3] Postuma R.B., Berg D., Stern M., Poewe W., Olanow C.W., Oertel W. (2015). MDS clinical diagnostic criteria for Parkinson’s disease. Mov. Disord..

[4] Goetz C.G., Tilley B.C., Shaftman S.R., Stebbins G.T., Fahn S., Martinez-Martin P. (2008). Movement disorder society-sponsored revision of the unified Parkinson’s disease rating scale (MDS- UPDRS): scale presentation and clinimetric testing results. Mov. Disord..

[5] Godinho C., Domingos J., Cunha G., Santos A.T., Fernandes R.M., Abreu D. (2016). A systematic review of the characteristics and validity of monitoring technologies to assess Parkinson’s disease. J. Neuroeng. Rehabil..

[6] J. Giuffrida (2014). U.S. Patent No. 8,679,038 B1. Washington, DC: U.S. Patent and Trademark Office.

[7] Yang C.-C., Hsu Y.-L. (2010). A review of accelerometry-based wearable motion detectors for physical activity monitoring. Sensors.

[8] Aquilonius S.M., Tiselius P. (1969). Measurement of rigidity and tremor in Parkinson’s disease. Acta Neurol. Scand..

[9] Elble R.J. (1986). Physiologic and essential tremor. Neurology.

[10] Haubenberger D., Abbruzzese G., Bain P.G., Bajaj N., Benito-León J., Bhatia K.P. (2016). Transducer-based evaluation of tremor. Mov. Disord..

[11] Marshall J. (1959). Physiological tremor in children. J. Neurol. Neurosurg. Psychiatr..

[12] Webster D.D. (1960). Dynamic measurement of rigidity, strength and tremor in Parkinson patients before and after destruction of mesial globus pallidus. Neurology.

[13] Wile D.J., Ranawaya R., Kiss Z.H.T. (2014). Smart watch accelerometry for analysis and diagnosis of tremor. J. Neurosci. Methods.

[14] Zhan A., Mohan S., Tarolli C., Schneider R.B., Adams J.L., Sharma S. (2018). Using smartphones and machine learning to quantify Parkinson disease severity: the mobile Parkinson disease score. JAMA Neurol..

[15] Papapetropoulos S., Katzen H.L., Scanlon B.K., Guevara A., Singer C., Levin B.E. (2010). Objective quantification of neuromotor symptoms in Parkinson’s disease: implementation of a portable, computerized measurement tool. Parkinsonss Dis..

[16] Scanlon B.K., Levin B.E., Nation D.A., Katzen H.L., Guevara-Salcedo A., Singer C., Papapetropoulos S. (2013). An accelerometry-based study of lower and upper limb tremor in Parkinson’s disease. J. Clin. Neurosci..

[17] Mancini M., Horak F.B., Zampieri C., Carlson-Kuhta P., Nutt J.G., Chiari L. (2011). Trunk accelerometry reveals postural instability in untreated Parkinson’s disease. Parkinsonism Relat. Disord..

[18] Zach H., Janssen A.M., Snijders A.H., Delval A., Ferraye M.U., Auff E. (2015). Identifying freezing of gait in Parkinson’s disease during freezing provoking tasks using waist-mounted accelerometry. Parkinsonism Relat. Disord..

[19] iPad [Device]. (2018). Cuperino, CA: Apple. Retrieved from: https://www.apple.com/ipad/.

[20] SMART Balance Master® [Device] (2018). Pleasanton, CA: Natus Medical Incorporated. Retrieved from: http://www.natus.com/index.cfm?page=products_1&crid=271&contentid=397.

[21] Ozinga S.J., Linder S.A., Alberts J.L. (2017). Use of mobile device accelerometry to enhance evaluation of postural instability in Parkinson disease. Arch. Phys. Med. Rehabil..

[22] Kinesia [Device] (2018). Clevelend, OH: Great Lakes NeuroTechnologies. Retrieved from: http://glneurotech.com/kinesia/contact/.

[23] Heldman D.A., Espay A.J., LeWitt P.A., Giuffrida J.P. (2014). Clinician versus machine: reliability and responsiveness of motor endpoints in Parkinson’s disease. Parkinsonism Relat. Disord..

[24] ADXL335 [Device] (2010). Norwood, MA: Analog [Devices].

[25] EVAL-ADXL335Z Device (2009). Norwood, MA: Analog [Devices].

[26] DI-710 Series [Device] (2014). Akron, OH: DATAQ Instruments, Inc.

[27] DATAQ Instruments, Inc (2014). DI-710 Series 16-channel USB or Ethernet Data Logger User’s Manual.

[28] WINDAQ [software] (2017). Akron, OH: DATAQ Instruments, Inc.

[29] G-Link-200-OEM Wireless Accelerometer Node [Device] (2017). Cary, NC: LORD Corporation. Retrieved from: http://www.microstrain.com/wireless/g-link-200-oem.

[30] LeMoyne R., Mastroianni T., Grundfest W. (2013). Wireless accelerometer configuration for monitoring Parkinson’s disease hand tremor. Adv. Parkinson’s Disease.

[31] World Medical Association (2016). WMA Declaration of Helsinki - Ethical Principles for Medical Research Involving Human Subjects. https://www.wma.net/policies-post/wma-declaration-of-helsinki-ethical-principles-for-medical-research-involving-human-subjects/.

[32] International Committee of Medical Journal Editors (ICMJE) (2015). Recommendations for the Conduct, Reporting, Editing, and Publication of Scholarly Work in Medical Journals. http://icmje.org/icmje-recommendations.pdf.

[33] Burkhard P.R., Langston J.W., Tetrud J.W. (2002). Voluntarily simulated tremor in normal subjects. Neurophysiol. Clin. [Clinical N].

[34] MATLAB [software] (2017). Natick, MA: Mathworks. Retrieved from: https://www.mathworks.com/products/matlab.html.

[35] MathWorks [software] (2018). fft. Natick, MA. Retrieved from: https://www.mathworks.com/help/matlab/ref/fft.html.

[36] MathWorks [software] (2018). Statistics and Machine Learning Toolbox. https://www.mathworks.com/products/statistics.html.

[37] MathWorks [software] (2018). Time-frequency Analysis with the Continuous Wavelet Transform. https://www.mathworks.com/help/wavelet/examples/time-frequency-analysis-with-the-continuous-wavelet-transform.html.

[38] Harrigan T., Brasic J.R., McKay G.N., Mills K.A., Bang J.Y.A., Hwang B.J. (2018). Wavelet Investigation of Accelerometry in Parkinson’s Disease [Abstract]. https://abstractsonline.com/pp8/#!/4649/presentation/38333.

[39] Harrigan T., Brasic J.R., McKay G.N., Mills K.A., Hwang B.J., Mishra C. (2017). Accelerometry in Parkinson’s Disease [Abstract]. http://www.abstractsonline.com/pp8/#!/4376/presentation/34603.

[40] Brasic J.R., McKay G.N., Hwang B.J., Harrigan T.P., Mishra C., Mills K.A. (2017). Quantitative Continuous Measurement of Movements in the Extremities of People with Parkinson’s Disease [Abstract]. http://www.abstractsonline.com/pp8/#!/4376/presentation/34605.

[41] Hwang B.J., McKay G.N., Harrigan T., Mishra C., Pantelyat A., Wong D.F., Brasic J.R. (2017). Test-retest of Instrumentation to Quantitatively Measure Movements of Parkinson’s Disease [Abstract]. http://www.abstractsonline.com/pp8/#!/4376/presentation/34604.

[42] Brasic J.R., McKay G.N., Harrigan T.P., Pantelyat A.Y., Mills K.A., Hwang B.J. (2018). Quantitative Continuous Measurement of Tremor [Abstract]. https://abstractsonline.com/pp8/#!/4649/presentation/38332.

[43] Kim H.B., Lee W.W., Kim A., Lee H.J., Park H.Y., Jeon H.S. (2018). Wrist sensor-based tremor severity quantification in Parkinson’s disease using convolutional neural network. Comput. Biol. Med..

[44] Holzenberger N., Du M., Karadayi J., Riad R., Dupoux E. (2018). Learning word embeddings: unsupervised methods for fixed-size representations of variable-length speech segments. Proc. Interspeech.

[45] Delrobaei M., Memar S., Pieterman M., Stratton T.W., McIsaac K., Jog M. (2018). Towards remote monitoring of Parkinson’s disease tremor using wearable motion capture systems. J. Neurol. Sci..

[46] Yang J.-L., Chang R.-S., Chen F.-P., Chern C.-M., Chiu J.-H. (2016). Detection of hand tremor in patients with Parkinson’s disease using a non-invasive laser line triangulation measurement method. Measurement.

[47] Aytürk Z., Yilmaz R., Akbostanci M.C. (2017). Re-emergent tremor in Parkinson’s disease: clinical and accelerometric properties. J. Clin. Neurosci..

[48] Niazmand K., Kalaras A., Dai H., Lueth T.C. (2011). Comparison of methods for tremor frequency analysis for patients with Parkinson’s disease. 4th International Conference on Biomedical Engineering and Informatics (BMEI).

[49] Elble R.J. (2005). Gravitational artifact in accelerometric measurements of tremor. Clin. Neurophysiol..

[50] Piro N.E., Baumann L., Tengler M., Piro L., Blechschmidt-Trapp R. (2014). Telemonitoring of patients with Parkinson’s disease using inertial sensors. Appl. Clin. Inform..

[51] Vicon [Device]. (2018). Yarnton, Oxford, UK: Vicon Oxford. https://www.vicon.com/motion-capture.

[52] National Center for Simulation in Rehabilitation Research (NCSRR) (2010). OpenSim. http://opensim.stanford.edu/work/index.html.

[53] Schlenstedt C., Muthuraman M., Witt K., Weisser B., Fasano A., Deuschl G. (2016). Postural control and freezing of gait in Parkinson’s disease. Parkinsonism Relat. Disord..

[54] Bekkers E.M.J., Van Rossom S., Heremans E., Dockx K., Devan S., Verschueren S.M.P., Nieuwboer A. (2018). Adaptations to postural perturbations in patients with freezing of gait. Front. Neurol..

[55] Barton J.E., Roy A., Sorkin J.D., Rogers M.W., Macko R. (2016). An engineering model of human balance control—part I: biomechanical model. J. Biomech. Eng..

[56] Vu L., Morgansen K.A. (2010). Stability of time-delay feedback switched linear systems. IEEE Trans. Automat. Contr..

